# The effect of provider- and workflow-focused strategies for guideline implementation on provider acceptance

**DOI:** 10.1186/1748-5908-4-71

**Published:** 2009-10-29

**Authors:** Mindy E Flanagan, Rangaraj Ramanujam, Bradley N Doebbeling

**Affiliations:** 1 VA Health Services Research & Development Center on Implementing Evidence-Based Practice, Roudebush VAMC, Indianapolis, Indiana, USA; 2 IU Center for Health Services & Outcomes Research, Regenstrief Institute, Inc., Indiana University, Indianapolis, Indiana, USA; 3 Owen Graduate School of Management, Vanderbilt University Nashville, Tennessee, USA; 4 Division of General Medicine & Geriatrics, Department of Medicine, Indiana University School of Medicine, Indianapolis, Indiana, USA

## Abstract

**Background:**

The effective implementation of clinical practice guidelines (CPGs) depends critically on the extent to which the strategies that are deployed for implementing the guidelines promote provider acceptance of CPGs. Such implementation strategies can be classified into two types based on whether they primarily target providers (*e.g.*, academic detailing, grand rounds presentations) or the work context (*e.g.*, computer reminders, modifications to forms). This study investigated the independent and joint effects of these two types of implementation strategies on provider acceptance of CPGs.

**Methods:**

Surveys were mailed to a national sample of providers (primary care physicians, physician assistants, nurses, and nurse practitioners) and quality managers selected from Veterans Affairs Medical Centers (VAMCs). A total of 2,438 providers and 242 quality managers from 123 VAMCs participated. Survey items measured implementation strategies and provider acceptance (*e.g.*, guideline-related knowledge, attitudes, and adherence) for three sets of CPGs--chronic obstructive pulmonary disease, chronic heart failure, and major depressive disorder. The relationships between implementation strategy types and provider acceptance were tested using multi-level analytic models.

**Results:**

For all three CPGs, provider acceptance increased with the number of implementation strategies of either type. Moreover, the number of workflow-focused strategies compensated (contributing more strongly to provider acceptance) when few provider-focused strategies were used.

**Conclusion:**

Provider acceptance of CPGs depends on the type of implementation strategies used. Implementation effectiveness can be improved by using both workflow-focused as well as provider-focused strategies.

## Background

Improving the quality of patient care requires the effective implementation of clinical practice guidelines (CPGs) that promote interventions of proven benefit and discourage ineffective interventions [1]. However, despite the growing availability of CPGs for a wide range of clinical conditions, the extent to which care providers adhere to these guidelines varies widely. Concerns about the widening gap between the scientific knowledge incorporated in CPGs and its utilization in practice have led to urgent calls for more widespread and effective implementation [2,3]. Currently, there is a limited evidence base to determine the most effective strategies for implementing CPGs [1].

The study reported here draws from a conceptual framework on organizational change [4,5] and the emerging work on implementation of CPGs [6-8] to investigate the following questions: How does providers' acceptance of a set of CPGs vary with the number of distinct strategies used for implementing that set of CPGs? Specifically, how does provider acceptance vary with the two commonly used yet conceptually distinct types of implementation strategies (*i.e.*, provider-focused versus workflow-focused implementation strategies)?

The effective implementation of CPGs results in changes in the practices of care providers such that providers routinely follow the CPGs whenever appropriate. However, the eventual practice change is preceded by a sequence of cognitive-affective changes wherein providers become aware of the guideline, agree with the guideline, decide to adopt the practices, and adhere to the guideline as appropriate [9]. Therefore, rather than solely target the eventual provider adherence, efforts to promote guideline-concordant behaviors must also target other facets of provider acceptance (*i.e.*, guideline-related knowledge and attitudes). It follows that an important task in implementation research is the identification and verification of factors that influence provider acceptance of CPGs.

In general, adherence to guidelines is better when implementation strategies involve a widespread approach that targets multilevel barriers (patient, provider, clinic, organization) to adherence [6]. By implementation strategies, we refer to specific interventions (*e.g.*, academic detailing, grand rounds presentations) that are deployed to provide the necessary information, knowledge, skills, incentives, and the infrastructure for adherence. The potential importance of multi-faceted implementation strategies is well recognized in implementation research [10-12]. However, prior studies have focused mostly on the effects of multi-faceted implementation strategies on adherence to guidelines but not provider acceptance. Yet, the organizational change literature suggests that implementation efforts provide early cues to people about the importance, usefulness, and ease of use of any proposed change [13]. In turn, these cues shape employee acceptance and attitudes toward change such as resistance or support and eventually to change-related behaviors. Applied to the implementation of CPGs, this suggests that different strategies used in a multi-faceted approach to implementation strategy might help shape provider acceptance of guidelines. Specifically, provider acceptance might be more favorable when more (and varied) rather than fewer implementation strategies are used.

However, studies that have investigated the effectiveness of multi-faceted implementation approaches in promoting adherence report mixed results. A recent review concluded that there might not be a dose response effect between the number of implementation strategies and overall effectiveness [7]. That is, increasing the number of implementation activities does not necessarily increase adherence to guidelines. In contrast, others report that multi-faceted approaches improve provider adherence [14,15]. One possible explanation for mixed findings may be that prior studies tended to treat all implementation strategies alike. However, from an organizational change perspective, implementation efforts typically target different organizational components, such as work processes, knowledge and skills of individuals, formal roles and responsibilities, and organizational culture [4,12,16]. Whereas efforts to change culture may require long-term change interventions, the other three components, which essentially refer to work and providers in the context of CPGs, can be changed through short-term interventions [16].

Implementation strategies can be broadly classified as being workflow-focused (*e.g.*, clinical reminders provide timely alerts about appropriate actions to change work flow) or provider-focused (*e.g.*, academic detailing, which provides information about evidence-based practices to the providers). Workflow-focused implementation strategies involve changes in the task context relevant to a specific set of CPGs. They seek to minimize contextual barriers to the adoption of CPGs and to put in place changes that facilitate the routinized adoption of CPGs. Examples of such strategies include changing the work processes to incorporate the guidelines, introducing reminder systems that provide timely alerts about CPGs, redefining the roles and responsibilities for non-physician staff in congruence with the requirements of the CPGs, and revising forms/procedures [17,18]. By contrast, provider-focused implementation strategies involve communication with the providers about a specific set of CPGs. They seek to minimize provider-level barriers to adherence and to create provider-level facilitators to adherence. Examples of such strategies include distributing information about guidelines to promote awareness, organizing grand rounds to educate providers, conducting clinical meetings to clarify the CPGs, and identifying champions who can informally encourage providers to adopt CPGs.

In principle, the two types of strategies are complementary and the effective implementation of a CPG requires both workflow-focused as well as provider-focused implementation strategies [16]. However, when multi-faceted implementation strategies vary in their mix of these two types of strategies, they may have different effects. In particular, given the same number of implementation strategies, a more balanced mix (large number of workflow-focused as well as provider-focused strategies) is more likely to lead to acceptance because they provide information about CPGs as well as facilitate adherence in the normal course of work.

The present study examined the implementation efforts for three evidence-based CPGs in the Veterans Health Administration (VHA) chosen for implementation as national performance measures: chronic obstructive pulmonary disease (COPD), chronic heart failure (CHF), and major depressive disorder (MDD) [19-21]. See Table 1 for details of these CPGs. These three guidelines were selected because they represent key performance measures for the VHA.

**Table 1:** VHA clinical practice guidelines for COPD, CHF, and MDD.

**Guideline**	**Release date**	**Content**
Chronic obstructive pulmonary disease (COPD)	April 2000	Recommendations for pharmacologic management, exacerbation, and patient education

Chronic heart failure (CHF)	February 2001	Recommendations for the pharmacologic management, physical examination, diagnosis, and nonpharmacologic management

Major depressive disorder (MDD)	May 2000	Recommendations for depression screening, assessment, and management

## Methods

### Sample

We sampled two populations for the present study: VHA quality managers and VHA providers. The sampling frame included 143 VAMCs with ambulatory care clinics. For the quality manager survey, the sampling frame included 416 quality managers, primary care administrators, or other personnel directly involved in CPG implementation in their facility's primary care clinics. Regional level quality managers were contacted to identify local quality managers and other primary care administrators involved in guideline implementation at each facility in their region. Local quality managers were then contacted to identify up to two other personnel involved in guideline implementation in their primary care clinics. The goal was to have at least one key informant from each facility who could knowledgeably answer questions about CPG implementation at their facility. The sampling, survey procedure, and instrument have been described in detail elsewhere [18,22].

At each facility, we sought to identify at least eight physicians, eight nurses, and four physician assistants (PA) and/or nurse practitioners (NP), if available. The primary goal of the parent project was to test organizational factors predicting guideline compliance. Rather than using individual provider data, these analyses were planned for facility level comparisons using aggregated data. Thus, the power calculations determined the number of responding facilities, and not the number of providers sampled at each facility. As a result, the number of providers sampled from each facility was intended to be adequate for understanding the implementation context. Additionally, the provider sample size per facility was based on pragmatic and budget concerns. The randomly selected provider sample included 4,621 providers. Providers who were retired, deceased, not appropriate for participation (*i.e.*, no longer providing primary care), or who had left the VAMC were removed (n = 394) resulting in a final sample of 4,227 providers, including 1,770 physicians, 1,643 nurses, and 814 PAs or NPs. PA and NP categories were collapsed because some facilities employed either PAs or NPs, but typically not both.

### Surveys

#### Overview

Survey development was based on a literature review, our existing instruments from prior studies of guideline implementation in community facilities and VAMCs [23-26], and findings from a multi-institutional focus group study of barriers and facilitators to CPG implementation [27]. The quality manager survey included items assessing the following: perceptions of provider support, knowledge, and adherence with CPGs; dissemination approaches; information about guideline implementation task forces and committees; contextual factors associated with implementation; facility culture, cooperation, and structure; and Veterans Integrated Service Network (VISN) leadership involved in implementation. The quality manager instrument has been extensively used and validated and is published [18,28,29].

The provider survey included items assessing the following: provider knowledge of and agreement with CPGs; provider support for CPGs; dissemination approaches; provider patient care workload; facility culture, cooperation, and structure; use of technology in implementation and maintenance; performance feedback; and facility's provision of information technology support. A demographic section was included in both surveys. The surveys utilized a Likert-type response scale that ranged from one, (not at all) to five (very great), wherever appropriate.

#### Implementation measures

CPG implementation was assessed separately for four medical conditions: diabetes mellitus, COPD, CHF, and MDD. Results for diabetes mellitus guideline implementation are not presented here because questions about provider acceptance for this guideline were not included in the survey.

The provider survey included questions about 14 distinct implementation strategies with respect to each guideline: academic detailing, clinical meetings, grand rounds, complete guideline, brief summary, pocket card summary, storyboards, forms created or revised, responsibilities of non-physicians changed, champion for the guideline, computer reminders, computer tools to document recommended services, teleconferences, and personal digital assistants. Using these responses, implementation strategies were categorized as either provider-focused (*e.g.*, clinical meetings, copy of the complete guideline) or workflow-focused (*e.g.*, forms created, responsibilities of non-physicians changed). See Table 2 for a list of provider-focused and workflow-focused implementation strategies. A count variable was computed for number of provider-focused implementation strategies (range zero to ten) and for number of workflow-focused implementation strategies (range zero to four).

**Table 2:** Operationalization of provider-focused and workflow-focused implementation strategies used in the study.

**Provider-focused implementation strategies**	**Workflow-focused implementation strategies**
**1**. Academic detailing	**1**. Forms created or revised
**2**. Clinical meetings	**2**. Responsibilities of non-physicians changed
**3**. Grand rounds presentations	**3**. Computer reminders
**4**. Complete guideline distributed to providers	**4**. Computer tools to document recommended services
**5**. Brief summary distributed to providers	
**6**. Pocket card summary distributed to providers	
**7**. Storyboards	
**8**. Champion for the guideline	
**9**. Personal digital assistants given to providers	
**10**. Teleconferences	

Similarly, the quality manager survey included a list of possible implementation strategies used for each guideline. Because quality managers had access to information about multiple implementation efforts, their reports were used as a facility-level report of implementation activity. With these data, a total count (versus two counts for the two types of strategies) of implementation strategies was created. We did not divide these reports to reflect the two categories of strategies because we were not interested in whether the facility report of these two implementation strategies influenced provider acceptance. Rather, our primary question was whether providers' acceptance varied with their awareness of the two types of strategies. Therefore, the total count variable was used as a covariate in statistical models to account for the climate in which implementation occurred. If more than one quality manager survey was completed for a facility, then the average number of implementation strategies was computed at that facility (for each CPG).

#### Provider acceptance of COPD, CHF, and MDD guidelines

Six items assessed provider's acceptance of each of the COPD, CHF, and MDD clinical guidelines. These items included questions about knowledge, agreement, relevance, and clarity of the COPD, CHF, and MDD guidelines. Also, two items asked whether the implementation approach at their facility improved their knowledge and delivery of best practices related to the guideline. The response set for each item was a five-point Likert-type scale that ranged from one (not at all) to five (very great). The mean value of each provider's responses to these six items with respect to each set of guidelines was used to measure the provider's acceptance. A low score indicated low acceptance and a high score indicated high acceptance.

#### Covariates

Covariates included provider gender (0 = male, 1 = female), provider year of birth, tenure in provider's current position (years), and general attitude toward CPGs. In addition, because some of the providers did not respond to all six items about provider acceptance, the number of items that a provider responded to was included as a covariate. We included a general attitude measure toward CPGs so that we could account for general dislike for CPGs and still test for differences among the three CPGs in provider acceptance. General attitude toward CPGs was computed as the mean of seven items: 'In general, in your opinion, to what extent are the VA clinical guidelines: 1) Likely to improve quality of care; 2) Oversimplified or 'cookbook' medicine; 3) A challenge to professional autonomy; 4) Good educational tools; 5) Too complex to work with; 6) Not used routinely because of workload;' and 7) 'In the past two years, when CPGs were implemented in your facility, to what extent: Were you hesitant to adopt guidelines?' (1 = not at all, 5 = very great). Responses to items two, three, five, six, and seven above were reverse scored so that a high score was indicative of a favorable attitude toward CPGs.

#### Procedures

In 2001, the quality manager survey was administered using a modified Dillman survey method. Initially, participants received an introductory letter, information summary, survey, and letters of support. Non-respondents received a reminder postcard one week later. Then, after two weeks, non-respondents received the entire survey packet again. Finally, follow-up phone calls were made to those who had not returned surveys. A similar procedure was followed in 2003 when the provider survey was administered. The University of Iowa Institutional Review Board and Iowa City VAMC research committees approved all survey packet materials and procedures.

### Statistical Analyses

First, descriptive statistics were generated for both the quality manager and provider surveys using facility averages. Next, using a multilevel framework, we related providers' reports of the number of implementation strategies within each class (provider- and workflow-focused) to their acceptance of the guideline. Due to the nested nature of these data (*i.e.*, providers were nested within facilities), using multilevel models (MLM; also known as hierarchical linear modeling) is appropriate [30]. In this situation, providers sampled from one facility will be more similar to one another than to providers from another facility. This similarity may be due to working under the same set of policies, with a specific patient population, or within the same cultural context. Thus, the assumption of independent observations is violated; and a typical linear regression model would lead to spurious conclusions [31].

MLMs are comprised of levels: level-one represents the individual (provider), and level-two represents the grouping variable (facility). For provider *i *at facility *j*, the following equation predicting outcome *y *is appropriate: y_ij _= α_j _+ β_j _x_ij _+ e_ij_. As shown, a provider's score on a certain outcome is partially due to hospital differences. The intraclass correlation (ICC) is one indicator of dependency among observations (providers) from the same hospital. For the present data, ICCs ranged from 0.03 to 0.07 indicating that 3% to 7% of the total variance is due to facility membership. The use of MLM in this case would provide benefits over using a fixed effects model.

In the present MLM analyses, provider-level predictors included count of provider-focused and workflow-focused implementation strategies, general attitudes toward all CPGs, provider gender, provider year of birth, provider tenure at the facility (in years), and number of items for the outcome variable that contained responses. The facility-level predictor included in the model was the average number of implementation strategies used for each guideline, as reported by quality managers at that facility. Additionally, the three hypothesized two-way interaction terms were included in the model. The outcome variable was provider acceptance of three CPGs. Separate models were tested for COPD, CHF, and MDD guidelines. All analyses were conducted using SAS, version 9.1 (Cary, NC).

## Results

Surveys were returned by 129 of the 143 VAMCs, representing a 90% response rate at the facility level. A total of 242 quality managers (58% response rate) and 2,438 providers (58% response rate) completed surveys. A single quality manager survey was returned from 42 facilities. However, follow-up indicated that most represented a single institutional response, reached by consensus among those surveyed. When multiple quality manager surveys from a facility were returned, the total number of implementation strategies reported was averaged to create a facility-level response. Using a general linear model for each condition, we tested the effect of the number of returned quality manager surveys (one, two, or three) on the total number of implementation strategies for that facility. No significant differences were detected.

Table 3 presents descriptive statistics for the participating VAMCs. The 129 facilities used in these analyses were widely distributed across the U.S. Notably, nearly half (45%) of the facilities were members of the Council of Teaching Hospitals. The mean size (number of beds) was 313; and, 16% of hospitals were located in rural areas. The mean age of providers surveyed was 50 years. The sample was more than half female (62%) and had an average employment tenure at the facility of about 11 years. Of those participating, 38% were physicians, 38% were registered nurses, and 13% were advanced registered nurse practitioners. Internal medicine was the most frequently reported clinical specialty (35%).

**Table 3:** Descriptive characteristics of the participating Veterans Affairs Medical Centers (n = 129) and providers (n = 2,438).

***Variable***	**Percent or Mean (std dev)**
**Facility Characteristics**	
Member of teaching council	45%
Total facility beds	313 (272)
Located in metropolitan area	84%
	
**Provider Characteristics**	
Age in years	50 (8.7)
Gender (% female)	62%
Duration employment at the facility	11 (8.4)
Professional Training	
Physician	38%
Registered Nurse	38%
Physician Assistant	7%
Advanced Registered Nurse Practitioner	13%
Licensed Practical Nurse	0%
Other	6%
Specialty	
Internal Medicine, General	35%
Internal Medicine, Specialty	17%
Family Medicine	9%
Geriatrics	7%
Other	35%
None	4%

As shown in Table 4, the two most used provider-focused implementation strategies were distributing the complete guideline and providing a brief summary of the CPG. This finding is consistent for COPD, CHF, and MDD. For workflow-focused strategies, creating computer reminders and using computer tools to document recommended services were the two most common strategies. Again, this pattern is consistent for COPD, CHF, and MDD guideline implementation.

**Table 4:** Percentage of providers (n = 2,438) reporting the use of 14 strategies for COPD, CHF, and MDD clinical guideline implementation.

**Implementation Strategies**	**COPD** **(%)**	**CHF** **(%)**	**MDD** **(%)**
**Provider-focused**			
Academic detailing	4.5	5.1	3.2
Clinical meetings	18.5	20.3	14.2
Grand rounds	12.3	13.9	8.9
Complete guideline	31.6	32.4	23.8
Brief summary	30.8	33.2	21.4
Pocket card	19.2	19.0	12.4
Storyboards	5.4	5.4	3.5
Champion for the guideline	17.0	18.5	12.4
Teleconferences	11.3	11.5	11.3
Personal digital assistants	1.2	1.4	1.1
			
**Workflow-focused**			
Forms created	11.0	11.4	10.0
Responsibilities of non-physicians changed	14.2	12.5	15.2
Computer reminders	26.9	25.0	26.7
Computer tools to document recommended services	20.6	19.4	20.0

Note: COPD = Chronic Obstructive Pulmonary Disease, CHF = Chronic Heart Failure, MDD = Major Depressive Disorder.

### Missing data

Due to concerns with missing data in the outcome variables potentially creating bias in our results, we compared respondents and non-respondents. We identified a few differences between these two groups. Non-respondents were older, female, and had a longer tenure at the facility. Additionally, non-respondents reported fewer implementation strategies (of both types). The covariates representing these differences are included in all models. Hence, the results from the multilevel models are considered valid.

### Type of implementation strategy related to acceptance of clinical guidelines

The type of implementation strategy (provider-focused versus workflow-focused) was related to provider acceptance of the COPD, CHF, and MDD guidelines. Both provider- and workflow-focused strategies were positively related to acceptance (Table 5). Additionally, the number of provider-focused strategies interacted with the number of workflow-focused strategies to predict acceptance.

**Table 5:** Parameter estimates from multi-level models predicting provider acceptance of COPD, CHF, and MDD guidelines.

**Predictor**	**COPD**	**CHF**	**MDD**
Intercept	2.80***	2.86***	2.33***
Gender (0 = male, 1 = female)	0.02	-0.04	-0.01
Year of birth Tenure (yrs)	-0.00 -0.01**	0.00 -0.00	0.00 0.00
General attitude toward CPGs	0.27***	0.27***	0.17***
Number of non-missing items included in outcome variable	0.32***	0.35***	0.26***
			
Number of facility-level implementation strategies	0.01	-0.00	-0.00
			
Number of provider-focused implementation strategies	0.15***	0.15***	0.19***
			
Number of workflow-focused implementation strategies	0.08***	0.09***	0.18***
			
Number of facility-level implementation strategies X provider-focused	0.00	0.00	0.00
			
Number of facility-level implementation strategies X workflow-focused	0.00	0.00	-0.01
			
Provider focused strategies X workflow focused strategies	-0.02***	-0.03***	-0.04***

Note: COPD = Chronic Obstructive Pulmonary Disease, CHF = Chronic Heart Failure, MDD = Major Depressive Disorder. * p < 0.05, ** p < 0.01, *** p < 0.001

**Figure 1:**
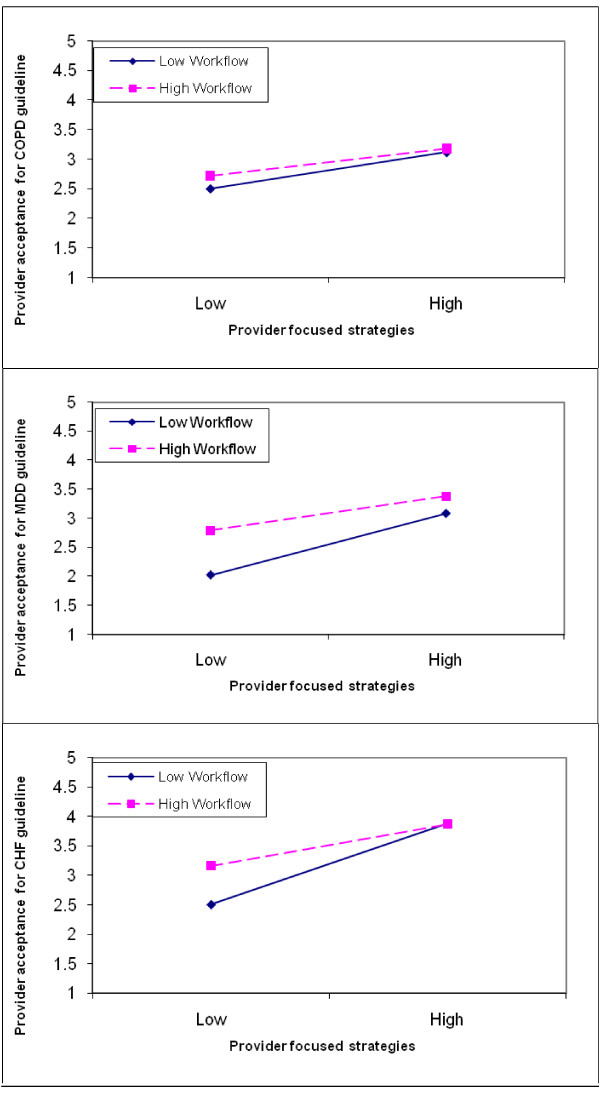
**(top panel) Provider-focused strategies X Workflow-focus strategies interaction for COPD guideline; (middle panel) Provider-focused strategies X Workflow-focus strategies interaction for CHF guideline; (bottom panel) Provider-focused strategies X Workflow-focus strategies interaction for MDD guideline**.

This interaction took a consistent form across the three sets of guidelines (Figure 1). To plot the interactions, 'low' and 'high' workflow-focused and provider-focused was defined based on 20% and 80% values, respectively, of the corresponding frequency distribution. These interactions suggested that multi-faceted implementations, which use both a high number of provider-focused and workflow-focused strategies, were positively related to acceptance of the COPD, CHF, and MDD guidelines. Additionally, these interactions suggested that even when relatively few provider-focused strategies were used, when paired with a high number of workflow-focused strategies, provider acceptance was similar to when a high number of provider-focused strategies was used.

To test whether the interaction terms improved each model, we compared the main effects only model to the full model (main effects and the three interaction terms). To conduct this comparison, we calculated the difference in -2 Log Likelihood values between the main effects only model and the full model for each condition. In all cases, the full model demonstrated a significant improvement.

## Discussion

Our findings suggest that the composition of the implementation strategies is critical for understanding provider acceptance of CPGs. Specifically, a higher number of provider-focused strategies was associated with acceptance of CPGs; providers from facilities endorsing a larger number of provider-focused strategies reported more acceptance of the guidelines. Significantly, this relationship was stronger in facilities that endorsed fewer workflow-focused strategies. In other words, provider-focused strategies may more strongly influence provider acceptance when such strategies are accompanied by fewer workflow-focused strategies. Overall, provider acceptance of CPGs for COPD, CHF, and MDD guidelines was lowest when neither type of strategy was used and highest when both strategies were used. Therefore, provider acceptance of CPGs was best predicted by provider-focused and workflow-focused implementation strategies when they were considered jointly.

Taken together, these findings offer one possible explanation for the inconsistent findings about the effectiveness of multi-faceted implementation strategies [7,14,15]. Previous studies, which provide mixed findings for the benefits of a higher number of implementation strategies, tended to treat all implementation strategies alike. However, as the current study indicates, the effects of the number of strategies may depend on the composition of strategies. For instance, the same number of provider-focused strategies may have different effects on provider acceptance, and, hence, on implementation depending on whether the facility additionally uses more or fewer workflow-focused strategies. In particular, if a facility already endorses a high number of workflow-focused strategies, the marginal benefits of a further increase in the number of provider-focused strategies may be limited. Therefore, future studies of multi-faceted implementation strategies should take into account differences across these strategies.

The findings from the study have important implications for efforts to implement CPGs. First, they suggest that, in general, using more distinct implementation strategies will help improve provider acceptance of guidelines. Second, they also highlight the important question facing implementation efforts--how best to use the available resources to elicit provider commitment to CPGs. This goal, in turn, requires selection of strategies that are directed at the providers as well as the workflow processes in which the CPGs must be embedded. Although our findings may be seen as implying that a large number of provider-focused strategies may be sufficient for improving acceptance, we would caution against such an interpretation. A mix of implementation strategies may be not only more efficient in using the available resources to improve acceptance but also more effective in promoting sustained adoption, which is the eventual goal of implementation efforts.

This study has several important strengths that should be emphasized. First, these data represent a large national sample of VAMCs and their efforts to implement CPGs across several chronic medical conditions. We considered a broad spectrum of organizational and provider-focused implementation strategies, based on the literature. Additionally, the multilevel aspect of this study adds strength to the design and interpretation. We obtained and compared data from multiple sources (*i.e.*, quality managers and providers), allowing us to consider simultaneous effects on provider acceptance of CPGs. Specifically, we were able to consider the implementation climate along with providers' view of implementation efforts as they influence a providers' acceptance of CPGs. With multiple data sources, our interpretations apply to more than one stakeholder group.

Some limitations of the current study design must be noted. Although we obtained a broad assessment of implementation efforts at the time of the survey, these data were cross-sectional. Hence, it does not permit us to draw causal inferences. While this study establishes associations between implementation efforts and provider acceptance, a longitudinal design will be needed to verify the relationship between provider acceptance and the implementation efforts used. Also, the current study measured the user attitudes toward the guidelines but not the extent to which the guidelines were being followed. Further studies are needed to verify the link between provider acceptance and adherence to the specific process measures from the guidelines considered in this study. Further research is also needed to determine which types of implementation strategies have the greatest sustained effect. Finally, the generalizability of the findings from this study to the target population may be limited due to the observed differences between respondents and non-respondents.

Our findings point to several other future research questions. What are the different classes of implementation strategies, in addition to the ones considered here, that might be relevant to understanding provider acceptance and guideline adherence? The current study considered some specific examples of provider-focused and workflow-focused strategies. What are some other specific strategies that fall under these categories? What are the social and cognitive processes that may help explain how the increased number of strategies and the joint reliance on different classes of implementation strategies lead to more provider acceptance of and adherence with guidelines?

## Conclusion

In conclusion, a multi-faceted implementation approach leads to provider acceptance of clinical guidelines. Implementation may be most effective in achieving provider buy-in when it relies on a mix of workflow-focused as well as provider-focused strategies.

## Competing interests

The authors declare that they have no competing interests.

## Authors' contributions

MEF was primarily responsible for research questions (for this manuscript), data analysis, and interpretation. RR contributed to the interpretation, drafting, and revision of the manuscript. BND was primarily responsible for study design, survey materials, obtaining funding support, data collection, and contributed to the interpretation, drafting and revision of the manuscript.
